# Downstaging after preoperative chemoradiation for locally advanced rectal cancer is associated with better survival than pathologic stage 0–1 disease treated with upfront surgery

**DOI:** 10.1007/s00384-023-04589-1

**Published:** 2024-01-08

**Authors:** Eli Kasheri, Avo Artinyan, Kimberly Oka, Ruoyan Zhu, Natalie Seiser, Mihran Shirinian, Moshe Barnajian, Jason Cohen, Joshua Ellenhorn, Yosef Nasseri

**Affiliations:** 1 Surgery Group LA, Los Angeles, USA; 2 Academic Surgical Associates, Glendale, USA; 3 Adventist Health Glendale, Glendale, USA; 4 Cedars-Sinai Medical Center, Los Angeles, USA

**Keywords:** Downstage, Survival, Locally advanced rectal cancer, NCDB

## Abstract

**Background and objectives:**

It is unknown how patients with locally advanced rectal cancer with significant response to preoperative radiotherapy/chemoradiotherapy fare relative to patients with true pathologic 0–1 disease undergoing upfront surgery. We aimed to determine whether survival is improved in locally advanced rectal cancer downstaged to pathologic stage 0–1 disease compared to true pathologic stage 0–1 tumors.

**Methods:**

A retrospective review of the National Cancer Database between 2004 and 2016 was conducted. Three groups were identified: (1) clinical stage 2–3 disease downstaged to pathologic stage 0–1 disease after radiotherapy, (2) clinical stage 2–3 disease not downstaged after radiotherapy, and (3) true pathologic 0–1 tumors undergoing upfront surgery. The primary endpoint was overall survival and was compared using Kaplan–Meier and multivariate Cox regression analyses.

**Results:**

The study population consisted of 59,884 patients. Of the 40,130 patients with locally advanced rectal cancer treated with preoperative radiation, 12,670 (31.5%) had significant downstaging (group 1), while 27,460 (68.4%) had no significant downstaging (group 2). A total of 19,754 had pathologic 0–1 disease treated with upfront resection (group 3). On Kaplan–Meier analysis, downstaged patients had significantly better overall survival compared to both non-downstaged and true pathologic stage 0–1 patients (median 156 vs. 99 and 136 months, respectively, *p* < 0.001). On multivariate analysis, downstaged patients had significantly better survival (HR 0.88, *p* < 0.001) compared to true pathologic 0–1 patients.

**Conclusions:**

Locally advanced rectal cancer downstaged after preoperative radiotherapy has significantly better survival compared to true pathologic stage 0–1 disease treated with upfront surgery. Response to chemoradiotherapy likely identifies a subset of patients with a particularly good prognosis.

## Introduction

The current standard of care for the treatment of locally advanced (clinical stage 2/3) rectal cancer (LARC) is neoadjuvant chemoradiation, followed by a total mesorectal resection (TME), with adjuvant chemotherapy [1]. The combination of multimodality therapy with a more standardized total mesorectal excision (TME) technique has resulted in improved outcomes, particularly with respect to decreased rates of local recurrence rate (LRR) [2–4].

Downstaging of rectal cancer with major clinical response (cMR) and complete clinical response (cCR) has received renewed attention because of the prospect of organ preservation strategies with “watch and wait” or local excision alone [5, 6]. Patients with LARC who have been downstaged after preoperative treatment and undergo TME are known to have improved survival compared with therapy-resistant tumors [4]. As a result, there has been a greater focus on improving downstaging with various forms of treatment including long-course neoadjuvant chemoradiation with delayed surgery and total neoadjuvant therapy [7, 8].

Although it is already known that downstaging significantly impacts survival, the prognosis associated with downstaging has been difficult to quantify, particularly in comparison to early-stage tumors treated with surgery alone [4]. Therefore, we sought to better understand the survival of LARC patients with significant downstaging following standard neoadjuvant chemoradiation and TME relative to patients with pathologic stage 0–1 disease treated with TME alone. We hypothesized that clinically staged LARC treated with upfront chemoradiation and downstaged to pathologic stage 0–1 disease has better survival compared to true pathologic stage 0–1 tumors treated with upfront surgery. 

## Materials and methods

### Study design

A retrospective review of the American College of Surgeons National Cancer Database (NCDB) was conducted from 2004 to 2016. The NCDB consists of data from over 1500 accredited Committee on Cancer facilities and is sourced from hospital registries.

### Patient selection

All patients over 18 years of age with non-metastatic rectal adenocarcinoma (site code C20.9) who underwent radical resection were included in this study. Rectal adenocarcinoma was identified using ICD-0–3 histology codes 8140–8147, 8260–8263, 8480–8481, and 8490. Radical resection was identified using FORDS codes 30–80 which included segmental/anterior resection (30–40), total proctectomy including abdominal perineal resection (50), and multiorgan resection including pelvic exenteration (70). Given the years that surgery was performed, TME was assumed.

Three distinct study groups were then described for the purposes of this study using the codes for clinical and pathologic TNM stage, radiation, and surgery-radiation sequence: (1) patients with clinical AJCC stage 2 or 3 disease (using NCDB clinical stage variable) who underwent preoperative radiotherapy and were downstaged to pathologic stage 0–1 (NCDB pathologic stage variable); (2) patients with clinical AJCC stage 2 or 3 disease, who underwent preoperative radiotherapy and not downstaged after radiotherapy (remainder pathology stage 2 or 3); and (3) AJCC pathologic stage 0–1 tumors, irrespective of clinical stage, who did not undergo radiation before surgery (true pathologic 0–1 tumors). The study population with patients categorized as above consisted of 59,884 patients.

### Statistical analysis

A descriptive analysis of the entire population was performed. Demographic factors including age, gender as well as clinical and pathologic factors such as Charlson/Deyo score (CDS), clinical stage, pathologic stage, margin status, histologic grade, number of harvested and positive lymph nodes, lymphovascular invasion (2010–2016), perineural invasion (2010–2016), and administration and sequence of chemotherapy received were described.

A univariate comparison of demographic, clinical, and pathologic factors by treatment group was performed using the chi-square test for categorical variables and one-way analysis of variance (ANOVA) for continuous variables. Overall survival was then compared among groups using the Kaplan–Meier method and the log-rank test. For overall survival, all deaths were included and patients alive at the last follow-up were censored. Multivariable Cox regression analysis was then performed to determine the independent association of the treatment group with overall survival. A specific missing data analysis was not performed. Given the large database nature of the study, missing data was assumed to be missing at random. The results of all statistical tests of significance were presented with appropriate measures of central tendency and variance. A *p*-value of < 0.05 was considered statistically significant.

## Results

A total of 59,884 patients with rectal cancer who met inclusion criteria were identified from the National Cancer Database. The demographics of the entire study population are shown in Table 1. The mean age of the population was 61.6 ± 12.7 years, and 61% of the population was male (Table 1). In the total cohort, 95.4% of patients had a margin-negative resection, and most patients (66.9%) received chemotherapy at some point during treatment. Of the 40,130 patients treated with preoperative radiotherapy for LARC, 12,670 (31.5%) had significant downstaging (group 1), while 27,460 (68.5%) did not have significant downstaging (group 2). A great majority of patients did not have significant comorbidity (CDS < = 1 94.3%), had low-grade disease (88.2%), and a significant majority (> 85%) had no lymphovascular invasion (LVI) or perineural invasion (PNI).

**Table 1:** Descriptive characteristics of the entire population

**(*n*** **= 59,884)**^**a**^	**No (%)**
**Age (years, mean ± SD)**	61.6 ± 12.7
**Sex** Male Female	36,344 (60.7%) 23,540 (39.3%)
**Lymph Nodes Harvested (mean ± SD)**	14.5 ± 9.48
**Charlson/Deyo score** 0 1 2 ≥ 3	45,792 (76.5%) 10,628 (17.7%) 2423 (4.0%) 1041 (1.7%)
**Lymphovascular invasion (2010–2016)** Absent Present	27,075 (85.2%) 4694 (14.8%)
**Margin status** Negative Positive	56,426 (95.4%) 2747 (4.6%)
**Perineural invasion (2010–2016) ** Negative Positive	30,486 (89.6%) 3530 (10.4%)
**Treatment group** LARC, downstaged to path 0–1 LARC, not downstaged to path 0–1 Path stage 0–1, upfront surgery	12,670 (21.2%) 27,460 (45.9%) 19,754 (33.0%)
**Chemotherapy received** No Yes	19,552 (33.1%) 39,605 (66.9%)
**Grade** Low grade High grade	46,622 (88.2%) 6210 (11.8%)
**Clinical stage** 0 1 2 3 Unknown	1067 (1.8%) 8195 (13.7%) 18,380 (30.7%) 22,505 (37.6%) 9737 (16.3%)
**Pathologic stage** 0 1 2 3	3095 (5.2%) 29,329 (49.0%) 12,793 (21.4%) 14,667 (24.5%)

^a^Missing data excluded for each variable

Table 2 summarizes the results of the univariate analysis of the group comparisons. Some of the comparisons are inherent to group selection, but they demonstrate the homogeneity of the study groups and the validity of the study. Importantly, in both groups who received preoperative radiation, > 90% of those patients also had chemotherapy initiated prior to definitive surgery, which indicates that most preoperatively treated patients likely received long-course chemoradiation.

**Table 2:** Univariate comparison of factors by treatment group

	**LARC w downstaging** **(*n* = 12,670)**	**LARC w/o downstaging** **(*n* = 27,460)**	**Path 0–1 upfront resection** **(*n* = 19,754)**	***p*-value**
**Age (years, mean ± SEM)**	60.5 ± 0.11	59.6 ± 0.07	65.2 ± 0.09	< 0.001
**Lymph nodes harvested (mean ± SEM)**	13.59 ± 0.08	14.94 ± 054	14.56 ± 0.08	< 0.001
**Sex** Male Female	8024 (63.3%) 4646 (36.7%)	17,138 (62.4%) 10,322 (37.6%)	11,182 (56.6%) 8572 (43.4%)	< 0.001
**Margin status** Negative Positive	12,329 (98.1%) 234 (1.9%)	24,733 (91.5%) 2299 (8.5%)	19,364 (98.9%) 214 (1.1%)	< 0.001
**Charlson/Deyo score** 0 1 2 3	9781 (77.2%) 2204 (17.4%) 487 (3.8%) 198 (1.6%)	21,807 (79.4%) 4409 (16.1%) 887 (3.2%) 357 (1.3%)	14,204 (71.9%) 4015 (20.3%) 1049 (5.3%) 486 (2.5%)	< 0.001
**Grade** Low High	9922 (91.5%) 924 (8.5%)	20,513 (84.6%) 3742 (15.4%)	16,187 (91.3%) 1544 (8.7%)	< 0.001
**Lymphovascular invasion**^**a**^ Absent Present	7060 (95.8%) 306 (4.2%)	12,036 (77.3%) 3527 (22.7%)	7979 (90.3%) 861 (9.7%)	< 0.001
**Perineural invasion**^**a**^ Absent Present	7915 (97.5%) 199 (2.5%)	13,815 (81.7%) 3099 (18.3%)	8756 (97.4%) 232 (2.6%)	< 0.001
**Clinical stage**^**b**^ 0 1 2 3 Unknown	0 0 6395 (50.5%) 6275 (49.5%) 0	0 0 11,455 (41.7%) 16,005 (58.3%) 0	1067 (5.4%) 8195 (41.5%) 530 (2.7%) 225 (1.1%) 9737 (49.3%)	–
**Pathologic stage**^**b**^ 0 1 2 3	1375 (10.9%) 11,295 (89.1%) 0 0	0 0 12,793 (46.6%) 14,667 (53.4%)	1720 (8.7%) 18,034 (91.3%) 0 0	–
**Chemotherapy initiation**^**b**^ Not received Preoperative Postoperative Unknown	230 (1.8%) 11,645 (91.9%) 151 (1.2%) 644 (5.1%)	568 (2.1%) 24,868 (90.6%) 545 (2.0%) 1479 (5.4%)	8754 (94.9%) 44 (0.2%) 250 (1.3%) 706 (3.6%)	–

^a^Data available after 2010

^b^Comparisons shown for descriptive purposes, no *p-*values given as these factors were part of treatment group selection

Patients who had significant downstaging after preoperative treatment (group 1) were equally likely to have started with clinical stage 2 or 3 disease (50.5 vs. 49.5%, respectively), while the majority of patients without a major pathologic response (group 2) had clinical stage 3 disease (58.3%).

Pathologic complete responders accounted for only 10.9% of patients with a significant response (group 1) and taken together with patients in group 2, this amounts to an overall pathologic complete response rate of 3.4%. While this is lower than reported elsewhere in the literature [9–11], the fact that the distribution of pathologic stage 0 and stage 1 patients are comparable in the downstaged group and the true pathologic 0–1 group (each about 10% and 90%, respectively) ensures a valid comparison between these study groups.

Additional findings on univariate comparison include a significantly older age in group 3 vs. groups 1 and 2 (65.2 ± 0.09 vs. 60.5 ± 0.11 and 59.6 ± 0.07 years, respectively, *p* < 0.001), a lower rate of LVI and PNI in group 1 vs. groups 2 and 3, (4.2% vs. 22.7% and 9.7%; and 2.5% vs. 18.3% and 2.6%, respectively, all *p* < 0.001) which is consistent with observed treatment response. As expected, non-downstaged (group 2) patients had the highest rate of margin positivity (8.5% vs. 1.9% and 1.1% for groups 1 and 3, respectively, *p* < 0.001).

**Fig. 1:**
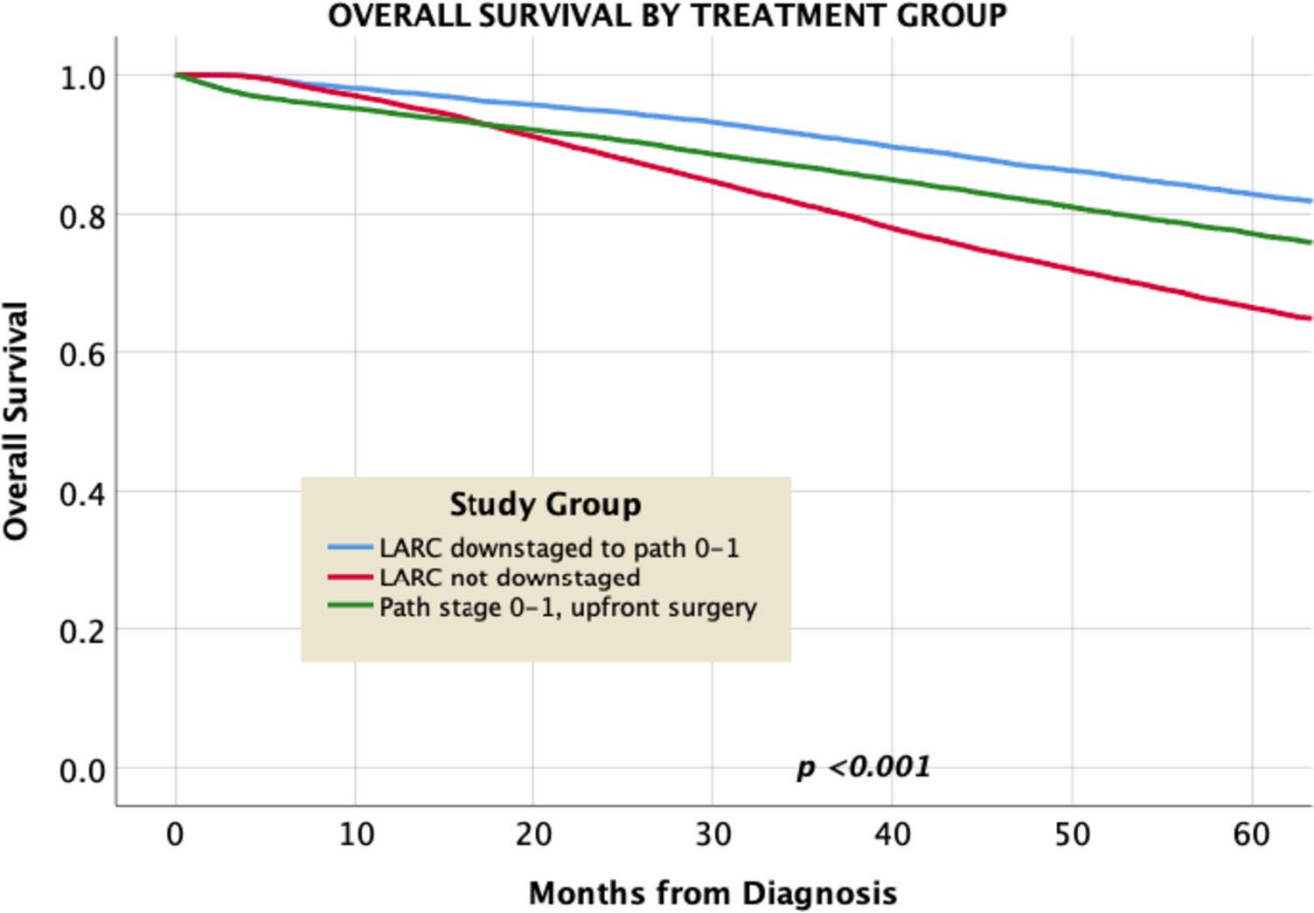
Kaplan–Meier analysis of overall survival

On overall Kaplan–Meier analysis (Fig. 1), downstaged patients (group 1) had significantly better overall survival compared to both non-downstaged and true pathologic stage 0–1 patients (median OS 156 vs. 99 and 136 months for groups 2 and 3, respectively, *p* < 0.001), with corresponding 5-year overall survival values of 83%, 66%, and 77%, respectively.

**Fig. 2:**
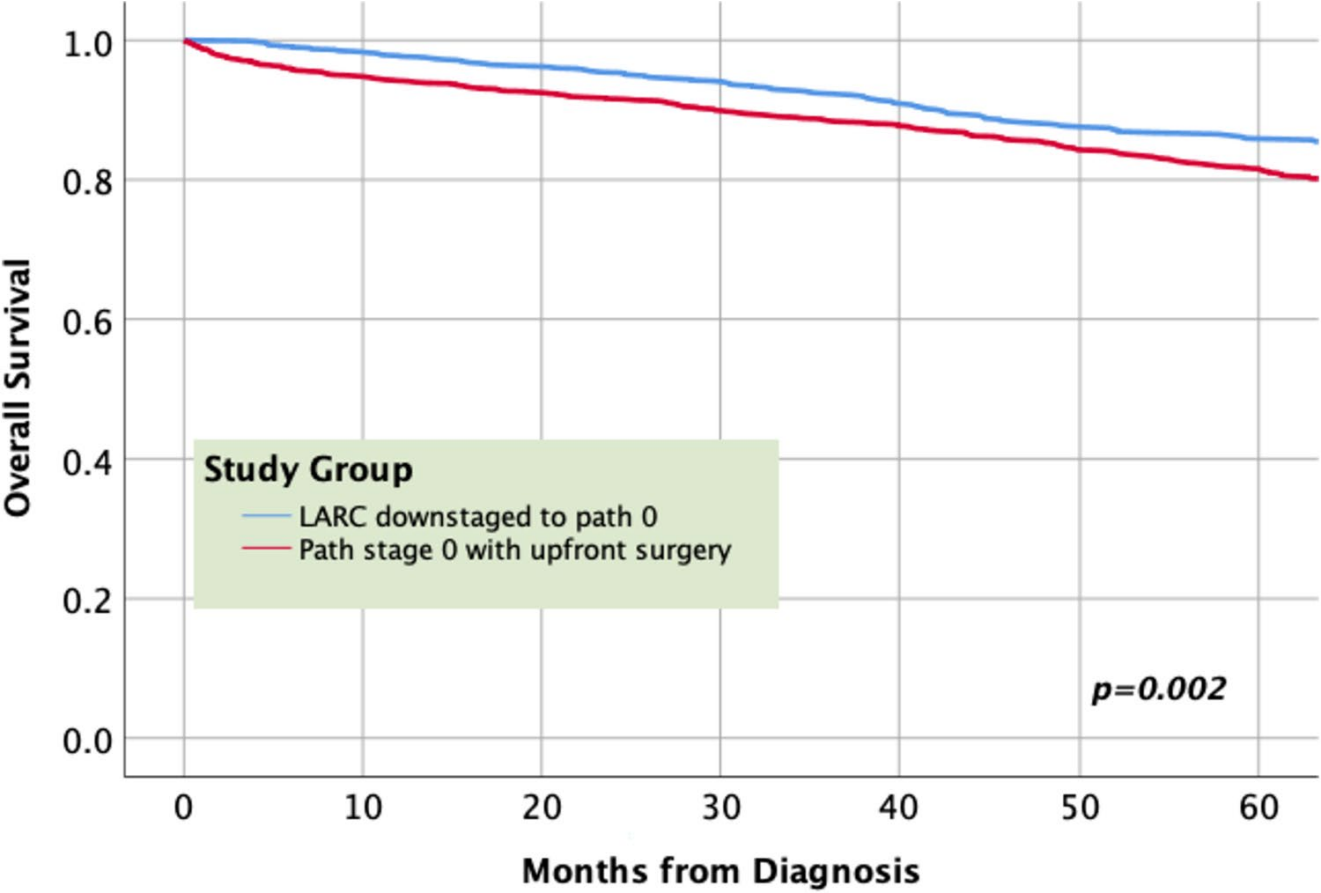
Stratified survival analysis for pathologic stage 0

**Fig. 3:**
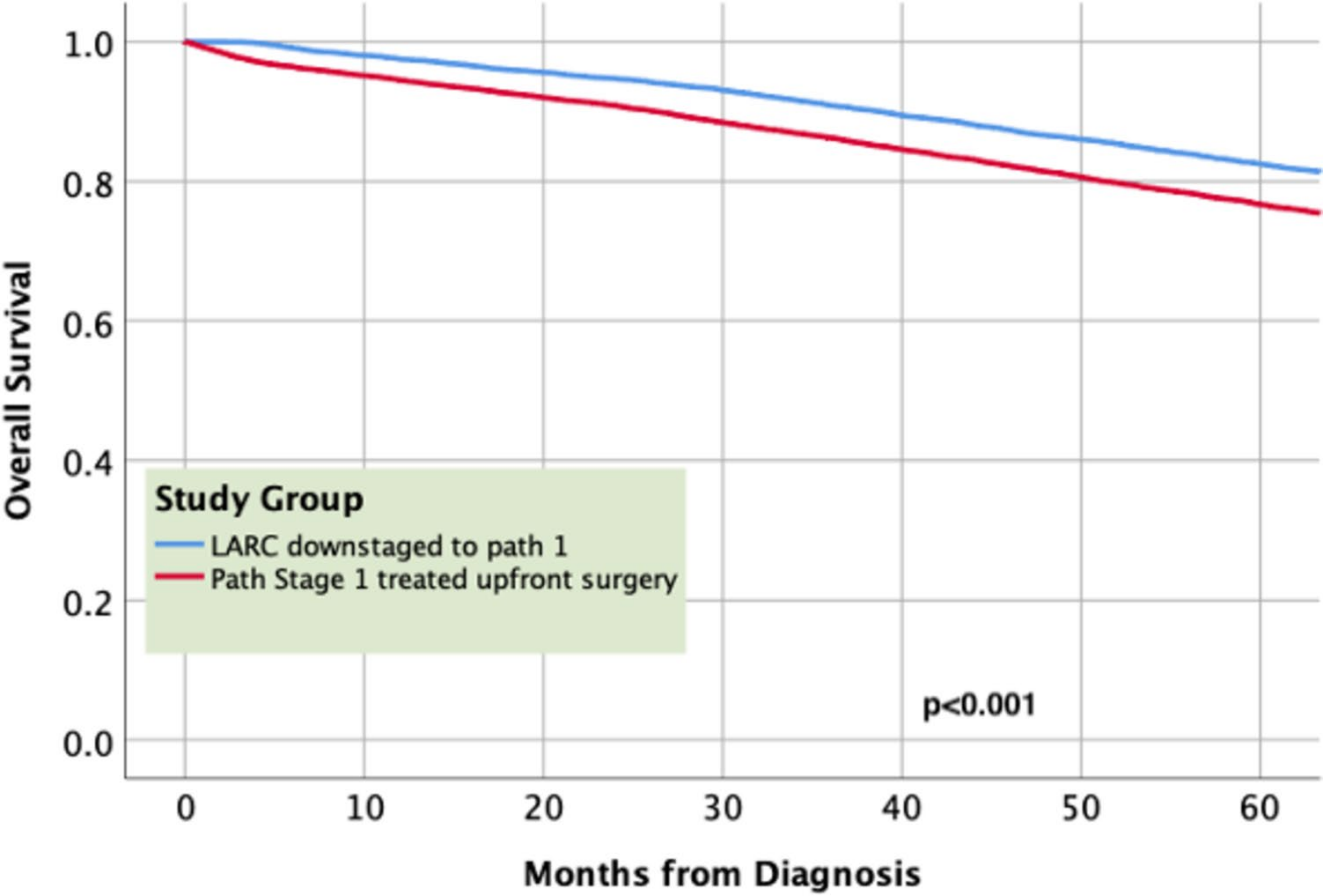
Stratified survival analysis for pathologic stage 1

On stratified survival analysis by pathologic stage, patients with preoperatively treated pathologic stage 0 and 1 disease continued to have improved survival compared to their untreated counterparts (Figs. 2, 3).

On multivariable analysis (Table 3), downstaged patients had significantly better survival (HR 0.88, *p* < 0.001) compared to true pathologic 0–1 patients, while non-downstaged patients had significantly worse survival (HR 1.78, *p* < 0.001).

**Table 3:** Multivariable Cox regression analysis of overall survival

	**Hazard ratio** **(95% CI)**	***p*-value**
**Treatment group** Path 0–I upfront surgery LARC downstaged to path 0–1 LARC not downstaged to path 0–1	ref 0.88 (0.83–0.93) 1.72 (1.66–1.79)	< 0.001 – < 0.001 < 0.001
**Age (years)**	1.04 (1.042–1.045)	< 0.001
**Sex** Male Female	ref 0.81 (0.78–0.84)	< 0.001 – < 0.001
**Charlson/Deyo score** 0 1 2 ≥ 3	ref 1.39 (1.34–1.45) 1.97 (1.84–2.11) 2.46 (2.22–2.73)	< 0.001 – < 0.001 < 0.001 < 0.001
**Lymph nodes harvested**	0.99 (0.992–0.996)	< 0.001
**Grade** Low grade High grade	ref 1.33 (1.27–1.39)	< 0.001 – < 0.001
**Margins** Negative Positive	ref 2.08 (1.95–2.22)	< 0.001 – < 0.001

## Discussion

We utilized the NCDB to evaluate the long-term survival of preoperatively treated and downstaged LARC patients relative to both patients who did not have a significant response as well as those with true pathologic stage 0–1 cancers treated with upfront surgery. In our study population, 31.6% of preoperatively treated LARC patients achieved significant downstaging to pathologic stage 0–1 disease. The complete pathologic response rate was only 3.4%, despite the fact that over 90% of patients were likely treated with long-course chemoradiation. Both the overall downstaging and pCR rate are significantly lower than other contemporary series utilizing upfront long-course chemoradiation or total neoadjuvant therapy [12, 13]. This difference is likely related to the variability in practice that is represented in large database studies, especially with respect to specific neoadjuvant regimens used as well as the time interval to surgery after completion of neoadjuvant therapy.

Consistent with existing literature, we demonstrated that patients with LARC after preoperative treatment have significantly better long-term survival compared with non-downstaged patients [14]. In our study, patients downstaged to pathologic stage 0–1 disease had even better long-term survival than primary pathologic stage 0–1 rectal cancer treated with upfront surgery. There is some variability in the literature with respect to the relative outcome of downstaged patients and their similarly staged counterparts treated with upfront surgery. For example, in a single institution series, Du et al. [15] did not demonstrate a significant difference in 5-year overall survival between ypstage I and pstage I patients. A similar study by Li et al. [16] found a significantly lower 5-year survival rate after propensity score matching in ypstage I patients (72.3% compared to 93.1% in the pstage I, *p* = 0.040). The differences between these studies and ours are unclear.

However, the fact that ypstage 0–1 patients have better outcomes than pstage 0–1 is not entirely unexpected. Ypstage 0–1 patients represent a biologically select group with better response to treatment and therefore potentially better survival, whereas pstage 0–1 patients represent an entirely unselected group. Within the context of a large database study and broad community practice in general, the unselected pstage 0–1 group may include patients who were understaged due to less than adequate mesorectal excision. In our anecdotal experience as a tertiary referral practice, inappropriate mesorectal excision has been among the most common reasons for understaging and resultant locoregional recurrence in patients with pathologic stage 1 rectal cancers.

Improved outcomes in downstaged patients are also directly and indirectly supported by findings from PRODIGE 23, EORTC, OPRA, and other organ preservation trials which collectively demonstrate (1) a potential outcome benefit for patients treated with upfront chemotherapy in some form and (2) very low systemic recurrence rates in LARC patients who have a locoregional response to treatment [8, 17, 18]. Given that treatment failures in our population of patients are likely to be predominantly systemic and that less failures were noted in patients with preoperative therapy, this lends support to aggressive treatment of LARC patients with upfront chemoradiation and chemotherapy both to increase CR rates and to potentially improve survival as in PRODIGE 23 [14].

Our study provides one of the largest and most up-to-date analyses of outcomes for LARC patients who have had major pathologic responses to radio/chemoradiotherapy. It gives further credence to the already wide agreement that neoadjuvant chemoradiotherapy leads to improvements in local control and suggests a potential survival benefit in patients treated with upfront chemotherapy as in PRODIGE 23 [14].

Our study contains biases and limitations typically associated with retrospective and large national database studies. First, a retrospective cohort study leads to a heterogeneous sample of patients in each group, causing greater variability in outcomes. Additionally, selection bias is likely in terms of follow-up data and may promote more favorable outcomes over their counterparts. However, this is assumed to be equal among each group. As far as the limitations of national databases, there is inherent variability in the clinical care of patients throughout their treatment. Differences in clinical staging modalities, neoadjuvant regimens, type of resection, and postoperative follow-up care vary by institution although this variability is a more realistic representation of real-world practice. The lack of clinical and oncological factors available prevents the assessment of relevant long-term outcome measures such as local recurrence, distant recurrence, and cancer-specific survival which would provide a more detailed assessment of primary outcomes. As our study consists of data from many different clinical settings, we believe our results reflect real-world outcomes.

## Conclusion

In conclusion, LARC patients with major pathologic downstaging after preoperative radiotherapy/chemoradiotherapy have significantly better survival compared to true pathologic stage 0–1 disease treated with upfront surgery. Although this finding may be the result of inconsistent TME in nationwide practice, it underscores the significance of treatment response to neoadjuvant therapy and suggests a survival benefit when chemoradiotherapy is routinely applied in a nationwide cohort.

## Data Availability

The data from this study are not publicly available.
